# Surface ECG interatrial block-guided treatment for stroke prevention: rationale for an attractive hypothesis

**DOI:** 10.1186/s12872-017-0650-y

**Published:** 2017-07-31

**Authors:** Antoni Bayés de Luna, Manuel Martínez-Sellés, Antoni Bayés-Genís, Roberto Elosua, Adrian Baranchuk

**Affiliations:** 1 Fundació Investigació Cardiovascular, ICCC, Hospital de Sant Pau, Barcelona, Spain; 2 Hospital General Universitario Gregorio Marañón. CIBERCV, Universidad Complutense, Universidad Europea, Madrid, Spain; 3 Hospital Universitari Germans Trias i Pujol, UAB, Barcelona, Spain; 4 Hospital del Mar Medical Research Institute, Barcelona, Spain; 5 Queen’s University, Kingston, ON Canada; 6 Servicio de Cardiología, Hospital General Universitario Gregorio Marañón, Calle Dr. Esquerdo 46, 28007 Madrid, Spain

**Keywords:** Interatrial block, Atrial fibrillation, Stroke, Risk

## Abstract

Atrial fibrillation (AF) is the most common sustained arrhythmia and is associated with stroke, cognitive impairment, and cardiovascular death. Some predisposing factors − as aging, diabetes, hypertension − induce and maintain electrophysiological and ultrastructural remodeling that usually includes fibrosis. Interatrial conduction disturbances play a crucial role in the initiation of atrial fibrosis and in its associated complications. The diagnosis of interatrial blocks (IABs) is easy to perform using the surface ECG. IAB is classified as partial when the P wave duration is ≥120 ms, and advanced if the P wave also presents a biphasic pattern in II, III and aVF. IAB is very frequent in the elderly and, particularly in the case of the advanced type, is associated with AF, AF recurrences, stroke, and dementia. The anticoagulation in elderly patients at high risk of AF without documented arrhythmias is an open issue but recent data suggest that it might have a role, particularly in elderly patients with structural heart disease, high CHA_2_DS_2_VASc (Congestive heart failure/left ventricular dysfunction, Hypertension, Age ≥ 75 [doubled], Diabetes, Stroke [doubled] – Vascular disease, Age 65–74, and Sex category [female]), and advanced IAB. In this debate, we discuss the association of surface ECG IAB, a marker of atrial fibrosis, with AF and stroke. We also present the rationale that justifies further studies regarding anticoagulation in some of these patients.

## Background

Atrial fibrillation (AF) is the most common sustained arrhythmia, and is currently considered a worldwide epidemic. AF usually occurs in patients with “atria at risk”, atria that have specific characteristics, including slow conduction. Fibrosis plays a key role in this process, especially in patients that present with AF in the context of advanced heart disease and aging [1]. Fibrosis usually is the consequence of a chain of events that are triggered by abnormal activation of the left atrium due to interatrial blocks (IABs) [2]. As recognized recently, AF is not necessary the main cause of stroke but rather is another important risk factor for stroke [3]. Interatrial conduction disturbances play crucial roles in the initiation of atrial fibrosis development and its associated complications.

## Interatrial block is an independent factor associated with atrial fibrillation, stroke, and cognitive impairment

**Fig. 1:**
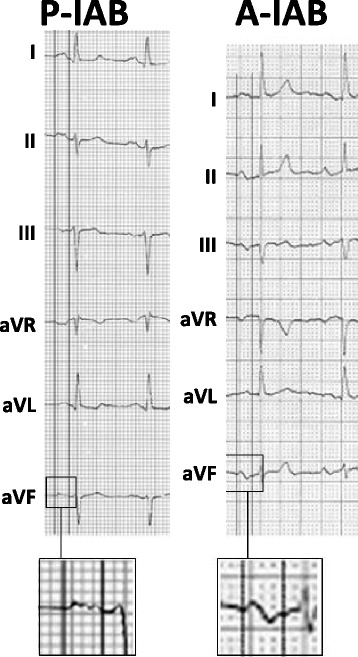
Example of partial interatrial block (P-IAB) (P-wave duration ≥120 ms, positive in leads II, III, and aVF) (*left*), and example of advanced interatrial block (A-IAB) (P-wave duration ≥120 ms with plus/minus morphology in leads II, III, and aVF) (*right*)

IAB is the most common and well-known block at the atrial level. IABs are classified as partial IABs (P-IABs) that are observed on the surface ECG as a positive P wave ≥120 ms, or as advanced IABs (A-IABs) manifested as a P wave ≥120 ms plus biphasic (±) morphology in leads II, III, and aVF (Fig. 1). In 2012, a consensus document was published that confirmed these criteria and stated that IAB was a separate entity from left atrial enlargement [4]. IAB is frequently found in the elderly, with a prevalence of 8% in septuagenarians and 25% in centenarians [5]. The prevalence is also high in patients with structural heart disease.

The biopsy tissue of patients with lone AF show abnormal atrial histology, as do the tissues of patients with paroxysmal AF [6]. Patients with lone AF present with different degrees of interatrial conduction disturbances and, compared with controls, present with more delayed and slower intra-atrial conduction and shorter effective refractory periods [7]. Patients with A-IABs have a higher incidence of AF than those with P-IABs [8], an association that is termed Bayes’ syndrome [9]. This association of IAB and AF has been confirmed in many studies performed in different settings, mainly by groups led by Spodick and Baranchuk [10, 11]. In a large cohort of unselected individuals, the incidence of AF was higher in the presence of A-IAB [12].

The association of IABs and embolic stroke has been demonstrated in hospitalized patients [13], in the general population [14], and in patients with high CHA2DS2-VASc scores [15]. Furthermore, Martinez-Sellés et al. [5] found that in centenarians, the presence of A-IAB is associated with previous stroke and cognitive impairment.

## How to measure atrial fibrosis?

Atrium fibrosis is a common feature in patients with AF, the causality of this association is not yet clarified but the association seems to be bidirectional: fibrosis increases the risk of AF that in turn induces fibrosis. In post-mortem samples, the extent of fibrosis and fatty tissue in the zones of the crista terminalis, Bachmann’s bundle region, and superior pulmonary veins is more extensive in patients with longer P-waves (*P* > 160 ms) [4]. Contrast-enhanced cardiac magnetic resonance (CMR) imaging may be the optimal non-invasive method for evaluating and quantifying left atrium fibrosis [16]. The amount of atrial fibrosis and the atrial strain rate, as detected by new echocardiographic techniques, correlates well with the likelihood of AF recurrence [16]. CMR imaging shows a higher mean value of fibrosis in patients with persistent vs. paroxysmal AF, yet the extent of fibrosis is not always related to the phenotype of AF. Large areas of fibrosis have been detected in some paroxysmal AF cases, while only mild fibrosis has been found in some persistent AF cases. Thus, the causal relation between AF duration and the extent of fibrosis requires further insight [16]. In patients with lone AF, the atrial substrate may progress despite successful AF ablation, indicating an independent progressive course in some cases of idiopathic atrial fibrotic cardiomyopathy.

It would be desirable to perform CMR or to use new echocardiographic techniques to determine the burden of atrial fibrosis to the full spectrum of patients at risk. In their absence, we recommend a careful analysis of the P-wave on surface ECG, which emerges as a cheap, easy and valuable surrogate of the atrial fibrotic process. Indeed, patients with A-IAB present with low atrial mobility due to the large amount of fibrosis and with reduced strain by speckle-tracking echocardiography [17]. This is important as left atrial deformation measured with speckle tracking echocardiography has been associated with AF recurrence [18]. Moreover, extensive atrial fibrosis assessed by late gadolinium enhancement CMR has been associated with advanced IAB [19].

## Should anticoagulation be used in some patients with advanced interatrial block before they have documented atrial fibrillation?

A-IAB is an independent risk factor for AF and embolic stroke, and there is evidence that this risk increases in the presence of a high CHA2DS2-VASc score and advanced age. Surface ECG IAB should be considered in the risk stratification of patients in sinus rhythm that are at high risk of stroke, even if they have no documented AF [20, 21]. Also, the bleeding risk of elderly patients that receive anticoagulation should be taken into account. HAS-BLED score for major bleeding is very useful for this risk assessment, although, in the case of AF the net clinical benefit of anticoagulation seems to exist in almost all patients, as the risk of ischemic stroke without anticoagulant treatment is higher than the risk of intracranial bleeding with anticoagulant treatment [22]. However, before anticoagulation therapy is indicated in patients with no documented AF, two steps should be taken. The first step is to perform a prospective international registry to confirm that IAB is associated with a high risk of AF and stroke in the elderly population with heart disease [22]. After showing the magnitude of this association, the second step is to perform a randomized control trial to test the benefits of anticoagulation in patients with heart disease, sinus rhythm, and A-IAB [23], probably adding as inclusion criteria other factor related with AF and stroke as advanced age [24] and atrial ectopy [25]. Our hypothesis also opens the door to other drugs, for instance as the renin-angiotensin-aldosterone system inhibition might have a role in the reduction of the risk of developing new onset AF [26], these medications might improve prognosis of patients with advanced IAB.

Finally, we would like to clarify that our hypothesis is based on data of patients without rheumatic heart disease. However, patients with IAB and rheumatic disease probably have an even higher risk of stroke as the burden of atrial fibrosis is noteworthy in patients with AF and rheumatic heart disease [27].

## Conclusions

The presence of IAB, which is easily identifiable on surface ECG, may be a marker of a chain of events that leads to AF and stroke. IAB is common in the elderly and may be considered as a surrogate marker of atrial fibrosis. Patients with heart disease and IAB might benefit from early anticoagulation even without documented AF. This hypothesis needs to be confirmed with larger studies.

## Abbreviations

- CHA_2_DS_2_VASc: Congestive heart failure/left ventricular dysfunction, Hypertension, Age ≥ 75 [doubled], Diabetes, Stroke [doubled] – Vascular disease, Age 65–74, and Sex category [female]
AF: Atrial fibrillation
CMR: Cardiac magnetic resonance
HAS-BLED: Hypertension, Abnormal renal and liver function, Stroke, Bleeding, Labile INRs, Elderly (≥65 years), Drugs or alcohol
IAB: Interatrial block
